# Complications of dual growing rod with all-pedicle screw instrumentation in the treatment of early-onset scoliosis

**DOI:** 10.1186/s13018-021-02267-y

**Published:** 2021-02-05

**Authors:** Mohammad Zarei, Mehdi Tavakoli, Ehsan Ghadimi, Alireza Moharrami, Ali Nili, Ali Vafaei, Seyed Saeed Tamehri Zadeh, Soroush Baghdadi

**Affiliations:** 1 Joint Reconstruction Research Center, Imam Khomeini Hospital, Tehran University of Medical Sciences, Keshavarz Blvd, Tehran, 1419733141 Iran; 2 Division of Orthopaedic Surgery, The Children’s Hospital of Philadelphia, Philadelphia, PA USA

**Keywords:** Early-onset scoliosis, Growing spine, Pedicle screw, Growing rod, Proximal junctional kyphosis

## Abstract

**Background:**

Treatment of early-onset scoliosis (EOS) is still a challenge to patients, families, and surgeons. Previous studies have indicated that EOS patients are at high risk for complications following growth-friendly surgery. This study was performed to evaluate the results and complications of all-pedicle screw dual growing rod instrumentation in the treatment of EOS.

**Methods:**

In an IRB-approved retrospective study, we searched the electronic medical records of our institution for all patients who underwent posterior spinal instrumentation for scoliosis between March 2014 and March 2017. Patients under the age of 10 at the time of surgery who were treated with a growth-friendly technique were then selected. Patients with incomplete records and less than 2 years of follow-up were excluded. Charts, operative notes, clinic visits, and radiographs were extracted. Radiographs were reviewed, and the main curve Cobb angle, thoracic kyphosis, pelvic tilt, pelvic incidence, sacral slope, and proximal junctional angles were measured. We specifically looked for any intra-operative or post-operative complications. Statistical analysis was performed to determine the risk factors of complications.

**Results:**

A total of 42 patients with a mean age of 4.8 ± 2.1 years (range, 1.5–8 years) were included in the final analysis. Patients were followed for a median of 34 months (range, 24–55). The major curve was corrected from a mean of 42.9° ± 10.7° to 28.8° ± 9.6° at the latest follow-up. Proximal junctional angles and thoracic kyphosis increased significantly during the follow-up period (both *P* values < 0.001). A total of 7 complications (17%) were observed. Four patients (10%) developed superficial surgical site infections, all of which resolved with antibiotics and one round of surgical debridement. Three cases (7%) of proximal junctional kyphosis (PJK) were encountered during the study period, none of which required revision surgery. Pre-operative thoracic kyphosis was the only significant risk factor for the development of PJK.

**Conclusions:**

Our findings suggest that in settings without access to magnetically controlled growing rods, dual growing rods with all-pedicle screw instrumentation is still a viable treatment strategy with comparable results and complications. The most common complications are infection and PJK, with the latter being associated with a larger pre-operative thoracic kyphosis.

## Introduction

The management of early-onset scoliosis (EOS), defined as a curve > 10° before the age of 10 years, is challenging [1]. While even the largest curves in adolescent idiopathic scoliosis (AIS) are often successfully treated with one-stage posterior spinal instrumentation and fusion, the same is not true in EOS [2]. The historical treatment strategies consisting of early fusion through anterior and/or posterior approaches were abandoned with a better understanding of the long-term effects. The adverse effects of early spinal fusion on the growth and maturation of the lungs and chest wall negate the benefits of creating a straight spine, with thoracic insufficiency syndrome and other complications increasing morbidity and mortality in affected patients. Growth-friendly constructs introduced a paradigm shift in the treatment of EOS by providing the ribs, chest wall, and spine complex the opportunity to grow while maintaining some degree of correction. The wide array of surgical non-fusion techniques can be loosely grouped in distraction-based and guided growth-based systems, with the former being utilized more commonly due to the familiarity of surgeons with the technique. While several distraction-based systems are available for use depending on the underlying pathology and patient characteristics, distraction of the ribs or spine with no to minimal fusion only at proximal and distal foundations is the guiding principle, while growth is supported by increasing the length of the construct in timely intervals. The goal is to promote pulmonary development and achieve a T1–T12 length of at least 18 cm at maturity in order to decrease long-term complications of early fusion. To this end, growing rod techniques have been unequivocally successful and have substantially decreased the rate of pulmonary complications of early fusion. The evolving treatment paradigm in EOS is one of the shining examples of how translational medicine has improved patient outcomes. When the inadequacy of the fusion techniques was evident, the joint efforts of biomedical researchers and surgeons led to the development of an array of novel techniques and devices that have substantially improved the standard of care; including but not limited to VEPTR (vertically expandable prosthetic titanium rib), MAGEC rods (magnetic expansion control), and vertebral tethers [3–7].

However, as expected from a dynamic construct, complications are not uncommon with growing rod instruments. More than half of EOS patients undergoing growing rod implantation will experience at least one complication [8]. Although most complications are minor, the high rates of unplanned return to the operating room are still a concern in growing rod constructs. Increased kyphosis of the vertebrae immediately cranial to the construct, or proximal junctional kyphosis (PJK), is a common complication with growing rod techniques, with an incidence of 20 to 45% depending on the definition and technique [8–13]. Several authors have attempted to compare the results and complications of different fixation strategies, including hooks, screws, wires, and hybrid constructs [14, 15]. However, controversy still exists regarding the ideal instrumentation strategy [16].

Despite the advent of magnetically controlled growing rods, they are still expensive and not available in developing countries. At our institution, we do not have access to magnetically controlled growing rods and have been treating EOS patients with dual growing rods with all-pedicle screw fixation with good results. The purpose of this study was to evaluate the results and complications of this technique, with an emphasis on post-operative proximal junctional angle (PJA) changes and PJK.

## Methods

After obtaining IRB approval, a retrospective chart review was performed to identify patients who underwent posterior spinal instrumentation between March 2014 and March 2017 at a referral center in Iran. From this database, patients with EOS (age < 10 years) who underwent growing rod instrumentation were selected. While we did not investigate the surgical indication, we typically perform growing rod instrumentation in EOS patients in whom the deformity is progressive despite cast or brace treatment, and also in cases with congenital scoliosis. Patients with incomplete records and those with less than 2 years of follow-up were excluded from this study.

Patient records were reviewed, and demographic data, underlying pathology, instrumented/fused levels, and the number and interval between lengthening surgeries were extracted. Complications of surgery, including intra-operative and post-operative, were extracted from operative and clinic notes. Infection, hardware failure (rod/screw breakage, pullout, or dislodgement), PJK, and neurological complications were of special interest. For patients who went on to receive a definite fusion during the study period, only pre-fusionfollow-ups were reviewed and included in this study.

All available radiographs were reviewed by a fellowship-trained spine surgeon, and the following measurements were done on pre-operative, post-operative, and latest follow-up posteroanterior and lateral radiographs: main curve Cobb angle, T1–T12 kyphosis (TK), pelvic incidence (PI), pelvic tilt (PT), and sacral slope (SS). Moreover, the upper instrumented vertebra (UIV) and the lower instrumented vertebra (LIV) were identified. The proximal junctional angle (PJA) was defined as the angle between the caudal endplate of the UIV and the cephalad endplate of the second supra-adjacent vertebrae above the UIV [15, 17]. A diagnosis of PJK was established in a consensus meeting between two authors if the PJA was > 10° [17, 18].

All surgeries were performed by a fellowship-trained spine surgeon with 10 years of experience in scoliosis surgery. The technique has been previously described by Akbarnia et al. [10]. In brief, the paraspinal muscles were only dissected to place the proximal, and distal foundations after the UIV and LIV are selected pre-operatively.All-pedicle screw constructs were used for all patients with fusion of proximal and distal instrumented levels. Subsequently, dual growing rods were placed intramuscularly between the two foundations. A custom-made brace was worn by patients at all times except for bathing for 6 months post-operatively. Lengthening of the rods was performed every 4 to 6 months for all patients, regardless of the curve progression, until sufficient spinal growth was achieved.

### Statistical analysis

Quantitative variables are expressed as mean ± SD and qualitative variables as number (percentage). Univariate logistic regression analysis was performed for each variable to determine the risk factors for the development of PJK. To compare the pre-operative and post-operative mean values, a non-parametric Wilcoxon signed-rank test was performed. A *P* value < 0.05 was considered statistically significant. All data were analyzed using IBM SPSS version 22.0 for Windows (Armonk, NY).

## Results

A total of 42 patients were included in the final analysis with a mean age of 4.8 ± 2.1 years (range, 1.5–8 years) at the time of the first operation, of which 22 (52%) were male. Patients were followed for a median of 34 months (range, 24–55) after their first surgery.

The etiology of EOS was idiopathic in 18 patients (43%), neuromuscular in 16 (38%), congenital in 5 (12%), and syndromic in 3 (7%). All patients underwent posterior instrumentation with all-pedicle screws and dual growing rods, and a transverse connector was also used in 25 patients (60%). The most common upper instrumented level was T2 in 18 patients (43%), followed by T3 in 11 (26%). The most common lower instrumented vertebra was L3 in 15 patients (36%), followed by L2 in 11 (26%). Patients underwent a median of 4 lengthening procedures (range, 3–7 operations).

**Fig. 1:**
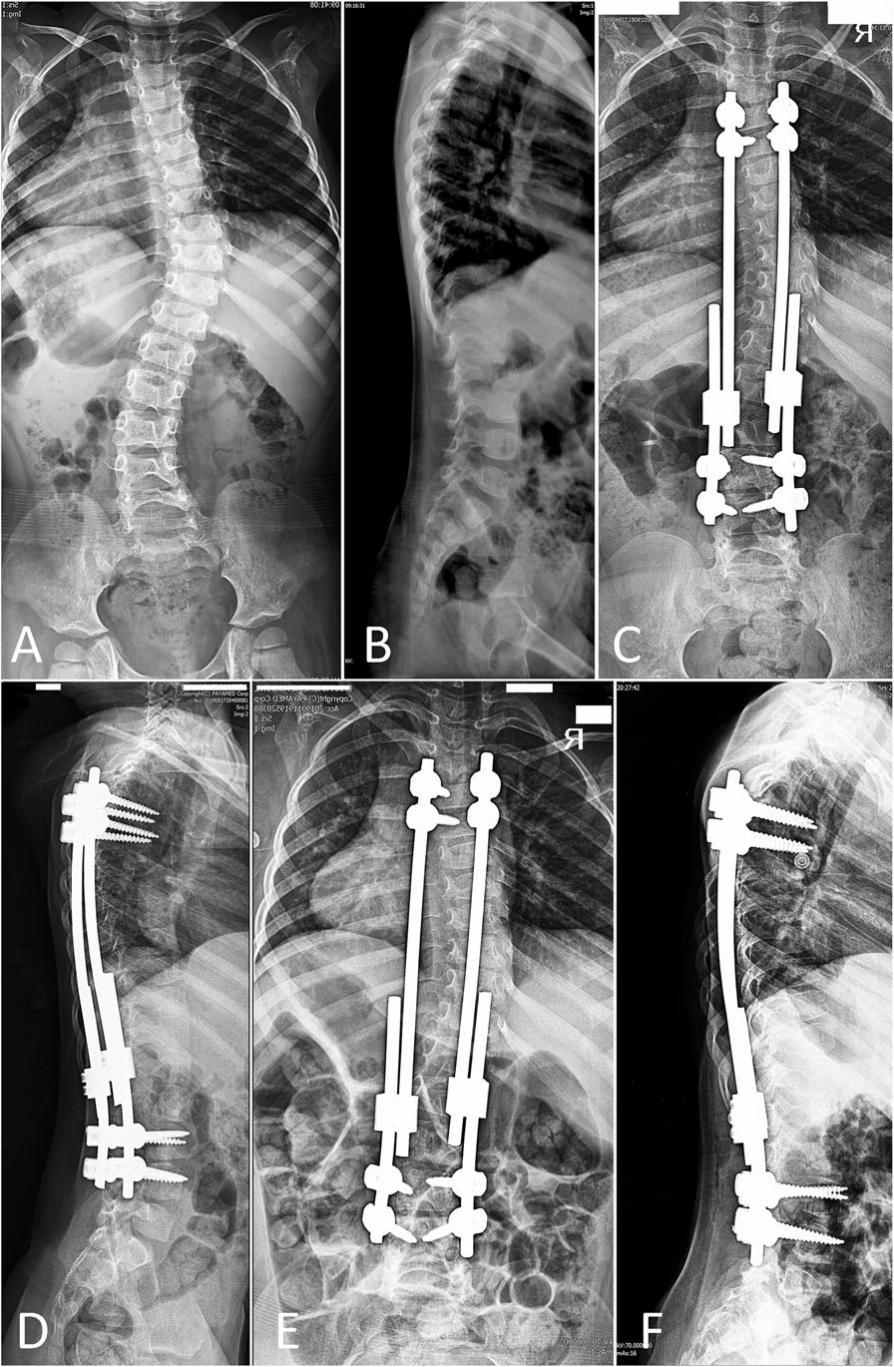
A five-year-old girl with idiopathic early-onset scoliosis (**a**-**b**) underwent dual growing rod instrumentation with all-pedicle screws (**c**-**d**). Latest follow-up radiographs (**e**-**f**) following four rounds of surgical lengthening reveals excellent correction with a proximal junctional angle of 5°. The patient is extremely active and happy with the results

The mean pre-operative Cobb angle of the major curve was 42.9° ± 10.7°, which was corrected to 28.8° ± 9.6° at the final follow-up (*P* < 0.001) (Fig. 1). Thoracic kyphosis increased significantly from pre-operative measurements to the latest follow-up by a mean of 12.6° ± 5.6° (*P* < 0.001). Proximal junctional angle also showed a significant increase during follow-ups. Changes in pelvic tilt, pelvic incidence, and sacral slope were not statistically significant. Post-operative changes are summarized in Table 1.

**Table 1:** Changes in the measurements from the pre-operative films to the latest follow-up. Values are presented as mean ± SD (range)

	Pre-operative	Latest follow-up	***P*** value
Major curve coronal Cobb angle	42.9 ± 10.7 (29–78)	28.8 ± 9.6 (10–53)	**< 0.001**
Thoracic kyphosis	30.3 ± 7.1 (15–48)	17.6 ± 5.6 (6–36)	**< 0.001**
Proximal junctional angle	5.4 ± 1.5 (3–10)	6.7 ± 3.0 (3–18)	**< 0.001**
Pelvic incidence	41.0 ± 9.3 (19–63)	43.1 ± 8.9 (23–64)	0.24
Pelvic tilt	14.9 ± 7.1 (4–34)	15.4 ± 5.7 (6–35)	0.43
Sacral slope	29.4 ± 6.6 (17–52)	30.1 ± 7.0 (20–52)	0.34

Significant *P* values are indicated in bold

## Complications

In total, we had seven complications (17%). Four patients (10%) developed superficial surgical site infection, all during the acute post-operative period. All were managed successfully with one round of surgical debridement, antibiotics, and primary closure. No cases of deep infection were observed. We also did not observe a case of hardware loosening or failure, as well as neurological impairments either during surgery or follow-up.

**Fig. 2:**
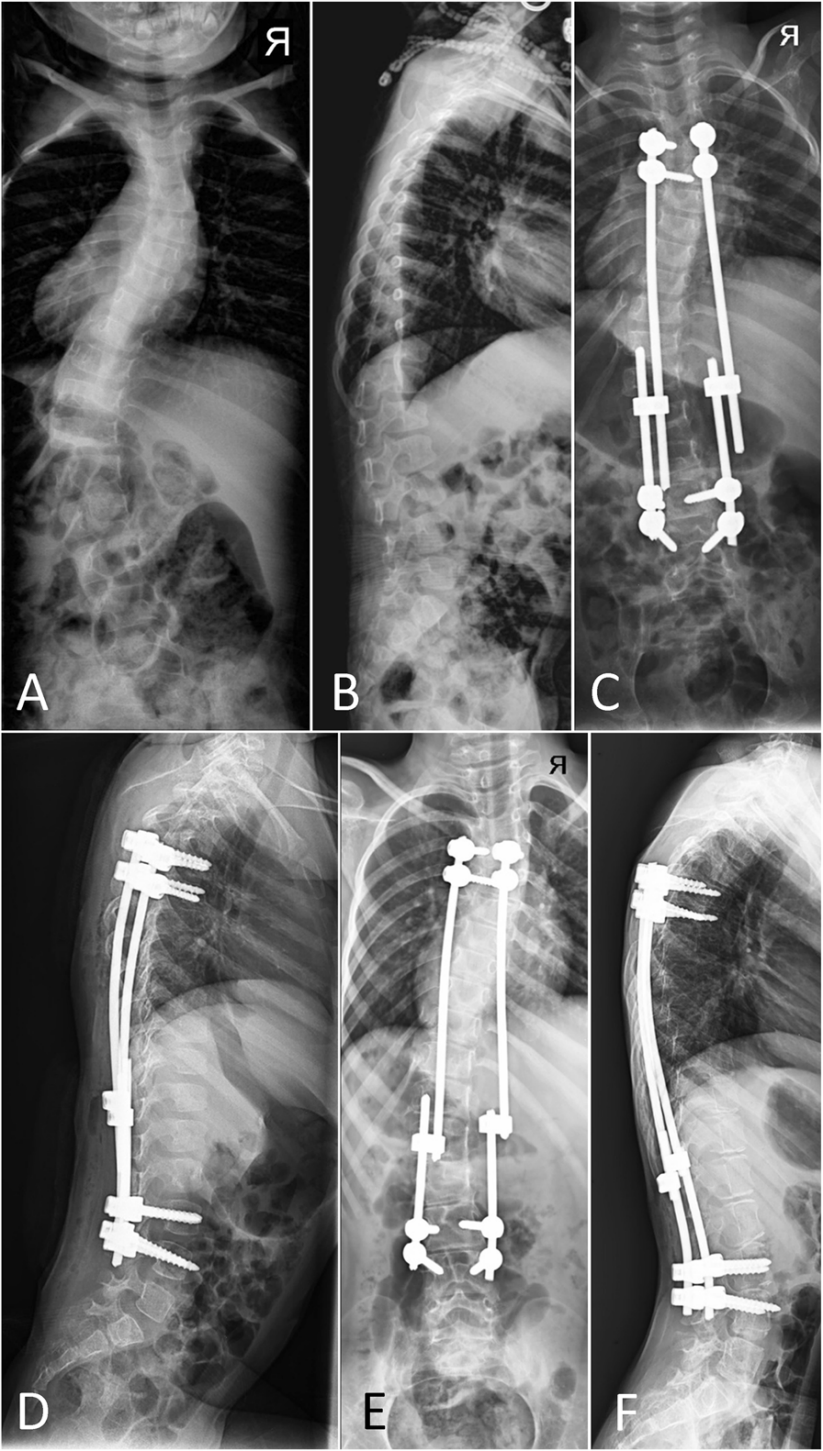
Proximal junctional kyphosis. Posteroanterior and lateral spine radiographs of a three-year-old boy with idiopathic early-onset scoliosis (**a**-**b**) who underwent dual growing rod instrumentation with all-pedicle screws (**c**-**d**). Three-year follow-up radiographs (**e**-**f**) reveal a proximal junctional angle of  18°, consistent with a diagnosis of proximal junctional kyphosis. The patient was asymptomatic and did not undergo revision

Three patients (7%) were diagnosed with PJK (Fig. 2) during the study period (with PJAs of 18°, 16°, and 15°). However, all three were managed non-operatively and were non-progressive. Patients with PJK had a significantly larger pre-operative TK (43.1° vs 29.0°, *P* = 0.001).

On univariate logistic regression analysis, age, gender, and scoliosis type were not significantly different between patients with and without PJK (*P* = 0.9, 0.6, and 0.8, respectively). Also, PI (*P* = 0.9), PT (*P* = 0.6), SS (*P* = 0.09), curve size (*P* = 0.2), correction degree (*P* = 0.6), UIV (*P* = 0.1), and LIV (*P* = 0.1) were not significantly different between patients with and without PJK.

## Discussion

Treatment of EOS is an evolving problem in pediatric orthopedics. While the final goal of treatment is the same as all spinal deformities, i.e., a straight spine and posture with a minimal number of operations and complications, there are unique aspects to the treatment of EOS. The surgeon should prioritize supporting the growth of the chest wall and lung maturation while preventing the progression of the curve. The traditional distraction-based guided growth system was modified and popularized by Akbarnia et al. [10] and is still the standard of care in the treatment of EOS.

There is an array of fixation strategies available for EOS, including hooks, sublaminar wires, and pedicle screws. While there is conclusive evidence that dual growing rods yield superior results than single rods [19–21], there is still a debate about the ideal hardware, especially in the proximal construct. Similar to the majority of institutions in the developing world, we do not have access to magnetically controlled growing rods due to their cost and have been using pedicle screws for all EOS surgeries. Therefore, this study was undertaken to evaluate the results of an all-pedicle screw dual growing rod system, and specifically to determine the risk of complications.

The growing rod technique, in contrast to a definite fusion, is a dynamic construct. The cyclic loads on the non-stiff growing rod construct, together with the inevitably smaller hardware due to the patient’s age, increase the risk of hardware-related complications. Furthermore, the patient has to undergo multiple lengthening procedures, which with the typical 4–6 months interval, would impose up to 10–15 surgical sessions on the patient, their caregivers, and the healthcare system. Therefore, complications should be anticipated, discussed with the patient/family, and close follow-up performed for early treatment of any complications. We had a total of seven complications (17%) in this study. While no neurological complications were observed, four superficial surgical site infections were encountered. All infections occurred after the initial surgery, and the following lengthenings were not complicated by infection. Additionally, all infections were treated with early debridement and antibiotics, and none progressed to a deep infection. The high rate of infection is thought to be the result of the underlying malnutrition EOS patients often have, which, combined with the small size of the child, increases the risk of skin dehiscence and infection [9].

Implant-related complications are also a substantial concern in non-fusion techniques. Myung and colleagues reported fewer implant-related complications in pedicle-screw constructs compared to hooks [22]. While we did not have patients treated with hooks, we did not observe an implant-related complication among our patients. Biomechanical studies have demonstrated the superiority of pedicle screws, with a foundation composed of four pedicle screws being the strongest construct in pullout tests [23].

PJK is another concern with a growing rod system, which could lead to cosmetic problems, progressive deformity, and implant failure. In line with previous reports, we found that the PJA increased significantly during treatment. However, only three cases (7%) of PJK were encountered, none of which required surgical treatment. The only significant predictor of PJK in the current study was pre-operative thoracic kyphosis. At the same time, the curve size, PI, PT, SS, UIV, and the correction achieved were not significant predictors of PJK. This is concordant with previous reports, which have shown that a pre-operative TK > 60° increases the risk of PJK [15]. Other authors have also found that the effect of pre-operative kyphosis is more pronounced when the UIV is distal to T2 [13, 24], which is why a UIV of T1 is desirable in hyperkyphotic patients, especially in neuromuscular curves.

The evidence is conflicting on whether all-pedicle screw constructs increase the risk of PJK. Shah et al. have found a higher incidence of PJK with screws [14], while Watanabe et al. did not find screws to significantly increase the risk of PJK [15]. There are, however, other preventable risk factors for PJK, including protecting the interspinal ligaments as much as possible. Also, contouring the rods into kyphosis at the proximal end of the construct prevents excessive forces on the spine immediately proximal to the proximal foundation [9].

We acknowledge the limitations of this study. While this study is a retrospective case series, the sample size is comparable to previous studies, while also having the advantage of being a homogeneous population treated by a single surgeon. Also, while we found a relatively low incidence of PJK, a larger study with a control group consisting of patients being treated with hooks or other constructs is needed to arrive at a definite conclusion. Moreover, with only three patients developing PJK, we were underpowered to discern the significance of some variables if present. Additionally, as some of our patients did not graduate from their growing rod construct and have not yet received a final fusion, we did not include follow-up data for those who did receive their final fusion.

## Conclusions

The findings of this study suggest that dual growing rods with all-pedicle screw instrumentation are viable treatment strategies in the treatment of EOS. The results and complications are comparable with magnetically controlled growing rods, which are expensive and not available in the developing world. The most common complications are infection and PJK, with the latter being associated with a larger pre-operative thoracic kyphosis. We are currently conducting trials on different fixation techniques and treatment strategies, which, combined with a larger sample size, will hopefully expand the findings of this study to different patient populations and underlying disorders.

## Data Availability

The datasets used and/or analyzed during the current study are available from the corresponding author on reasonable request.

## Abbreviations

EOS: Early-onset scoliosis
AIS: Adolescent idiopathic scoliosis
PJA: Proximal junctional angle
PJK: Proximal junctional kyphosis
TK: Thoracic kyphosis
SS: Sacral slope
PI: Pelvic incidence
PT: Pelvic tilt
UIV: Upper instrumented vertebra
LIV: Lower instrumented vertebra
